# Amyloid pathology induces dysfunction of systemic neurotransmission in aged APPswe/PS2 mice

**DOI:** 10.3389/fnins.2022.930613

**Published:** 2022-08-05

**Authors:** Se Jong Oh, Namhun Lee, Kyung Rok Nam, Kyung Jun Kang, Sang Jin Han, Kyo Chul Lee, Yong Jin Lee, Jae Yong Choi

**Affiliations:** ^1^Division of Applied RI, Korea Institute of Radiological and Medical Sciences, Seoul, South Korea; ^2^Radiological and Medico-Oncological Sciences, University of Science and Technology (UST), Seoul, South Korea

**Keywords:** positron emission tomography, Alzheimer’s disease, APPswe/PS2, beta amyloid, neurotransmitter

## Abstract

This study aimed to investigate how amyloid pathology affects the functional aspects of neurotransmitter systems in Alzheimer’s disease. APPswe/PS2 mice (21 months of age) and wild-type (WT) mice underwent positron emission tomography (PET) and magnetic resonance spectroscopy (MRS). First, we obtained ^18^F-FDG and ^18^F-florbetaben PET scans to evaluate neuronal integrity and amyloid pathology. Second, ^18^F-FPEB and ^18^F-FMZ PET data were acquired to assess the excitatory-inhibitory neurotransmission. Third, to monitor the dopamine system, ^18^F-fallypride PET was performed. Amyloid PET imaging revealed that radioactivity was higher in the AD group than that in the WT group, which was validated by immunohistochemistry. In the cortical and limbic areas, the AD group showed a 25–27% decrease and 14–35% increase in the glutamatergic and GABAergic systems, respectively. The dopaminergic system in the AD group exhibited a 29% decrease in brain uptake compared with that in the WT group. A reduction in glutamate, *N*-acetylaspartate, and taurine levels was observed in the AD group using MRS. Our results suggest that dysfunction of the neurotransmitter system is associated with AD pathology. Among the systems, the GABAergic system was prominent, implying that the inhibitory neurotransmission system may be the most vulnerable to AD pathology.

## Introduction

Alzheimer’s disease (AD) is a progressive neurodegenerative disorder and the most common cause of dementia. The pathological hallmarks of AD are the deposition of extracellular senile plaques (Aβ) and intracellular neurofibrillary tangles in the brain, which act as neurotoxins, causing synaptic loss, neuronal cell death, and neuroinflammation (Serrano-Pozo et al., 2011). Sustained neurodegeneration in patients with AD manifests as various clinical symptoms, including memory loss, cognitive impairment, and behavioral abnormalities (Tarawneh and Holtzman, 2012). According to the World Health Organization, approximately 50 million people are affected by AD, with nearly 10 million new cases reported annually (WHO Guidelines, 2019). As the aging population increases, the prevalence of AD is expected to significantly increase. Although efforts have been made over the past few decades to conquer AD, no efficient therapeutic drug has been established to prevent disease progression or restore brain functions. This highlights the need to expand our understanding of AD pathophysiology.

Neuroimaging enables the non-invasive evaluation of structural and functional changes in the brain. Typically, patients with AD show atrophy in the medial temporal lobe and a significant reduction in the rate of brain glucose consumption. Therefore, regional atrophy and hypometabolism are promising diagnostic imaging biomarkers (Kas et al., 2020). In terms of disease cascades, functional changes occur prior to anatomical changes, and the use of functional molecular imaging for the early diagnosis of AD is ideal. Positron emission tomography (PET) provides information on biological processes at the molecular level and is a functional molecular imaging technique (Lu and Yuan, 2015). Although PET has been recently applied in the diagnosis of AD using pathology-specific radiopharmaceuticals, the neurobiological characteristics of AD remain poorly understood (Rowley et al., 2020). In other words, most of the previous studies focused on the amyloid and tau burden with AD progression, but the question remains as to the effect of these pathological changes on the functional aspect of the neurotransmitter systems.

The involvement of excitatory and inhibitory neurotransmission systems is over 80%, thereby making these systems vulnerable to neurotoxins. The principal excitatory and inhibitory neurotransmitters are glutamate (Glu) and γ-aminobutyric acid (GABA), respectively. The equilibrium between these two neurotransmitters is linked to synaptic plasticity and normal brain function (Foster and Kemp, 2006). Dysfunction of these systems is associated with psychiatric disorders (Sarawagi et al., 2021). In addition, the dopaminergic system plays a vital role in synaptic plasticity and is considered a key modulator of emotion and cognition (Pan et al., 2019). A comprehensive evaluation of the neurotransmission system is required to understand AD pathophysiology because each system is interconnected.

However, previous studies have focused on individual neurotransmissions and not on systemic effects in live organisms. To address this gap, we evaluated changes in neuronal integrity and the degree of pathological damage using glucose or amyloid PET. Subsequently, changes in the excitatory-inhibitory and dopamine systems were assessed to observe reward circuits using glutamate, GABA, and dopamine PET. After finishing the imaging studies, we assessed the histopathology of the control and AD mice. Here, we used aged APPswe/PS2 mice as an AD animal model because this strain is known to have a significant amyloid pathology from 19 months of age (Brendel et al., 2015).

## Materials and methods

### Animals

In a previous amyloid PET study using various transgenic mice, including APPswe/PS2, APP/PS1dE9, G394A, and APPswe, amyloid deposition was detected only in APPswe/PS2 mice at 8 months of age using the cortical standardized uptake value (SUVR) relative to the cerebellum. This SUVR is highly correlated with the pathology of plaque deposition (Brendel et al., 2015). Therefore, this mouse model was selected for the present study. Double APPswe/PS2 transgenic [strain name: C57BL/6-Tg(NSE-hPS2*N141I); Tg(NSE-hAPPsw)Korl, male, AD, *n* = 6] and wild-type mice (B6C3F1, WT, male, *n* = 6, 8 months old), were provided by the National Institute of Food and Drug Safety Evaluation (NIFDS, Cheongju, South Korea). The care, maintenance, and treatment of animals in this study followed protocols approved by the Institutional Animal Care and Use Committee (permission number: kirams2020-0070), and experiments involving animals were conducted according to the Guide for the Care and Use of Laboratory Animals published by the US National Institutes of Health. The living chambers of the mice were automatically controlled with a 12-h light/dark cycle at a temperature of 22 ± 3°C and relative humidity of 55 ± 20%. Sterilized rodent diet and purified tap water were provided *ad libitum*.

### Study protocol

The experiments included two groups, the WT and AD groups, and the animals received care for 13 months (8–21 months of age). To examine abnormalities in the neurotransmission system, various PET scans (^18^F-FDG, ^18^F−florbetaben, ^18^F-FPEB, ^18^F-flumazenil, and ^18^F-fallypride) were obtained from the same mouse at 21 months of age. For anatomical information acquisition and magnetic resonance spectroscopy (MRS) analysis, magnetic resonance (MR) images were sequentially obtained for the same individuals. After the imaging studies were completed, all animals were sacrificed, and their brain tissues were prepared. Immunohistochemistry experiments were performed to quantify Aβ_42_ levels in the brain (21 months of age).

### Radiosynthesis of radiotracers

^18^F-FDG was provided by the Radiopharmaceutical Production Team of KIRAMS, and ^18^F-florbetaben was purchased (Ci-Co Healthcare Co., Ltd., Seoul, South Korea). Other radiotracers, ^18^F-FPEB, ^18^F-flumazenil, and ^18^F-fallypride, were synthesized according to previously described procedures (Seok Moon et al., 2010; Moon et al., 2014; Kang et al., 2021). The radiochemical purity of all radiotracers at the end of the synthesis was 99%.

### Positron emission tomography imaging

To observe the neurological changes in AD mice, glucose metabolism (^18^F-FDG), amyloid (^18^F-florbetaben), glutamate (mGluR5, ^18^F-FPEB), GABA (GABA_A_,^18^F-flumazenil), and dopamine (D_2_R,^18^F-fallypride) PET scans were performed using a small animal PET/CT scanner (nanoScan^®^, Mediso Medical Imaging Systems, Budapest, Hungary). Mice were anesthetized with 2.5% isoflurane in oxygen, and 8.4 ± 0.7 MBq of radiotracers in 200 μL of saline was intravenously injected *via* the tail vein. In the case of ^18^F-FDG, static PET scanning was performed 40–60 min post-injection (p.i., Mosconi, 2013). For the other tracers, dynamic PET scanning was performed for 50 min with 25 frames (14 × 30 s, 3 × 60 s, and 8 × 300 s). The image scans were acquired in an energy window of 400–600 keV. All images were reconstructed using the three-dimensional ordered subset expectation maximization (3D-OSEM) algorithm with four iterations and six subsets. For attenuation correction and anatomical reference, micro-CT imaging was conducted immediately after PET, using 50 kVp X-ray voltage at 0.16 mAs.

### Magnetic resonance imaging

To define the anatomical volumes of interest (VOIs), MR scans were obtained on a 31-cm horizontal-bore Agilent 9.4-T scanner (Agilent Technologies, Santa Clara, CA, United States) using a 2-channel array mouse head surface coil (Rapid Biomedical GmbH, Rimpar, Germany). The image parameters for the turbo spin echo (TSE) 3D T2-weighted image were as follows: repetition time (TR) = 2500 ms; echo time (TE) = 7.45 ms; FOV = 20 mm × 20 mm × 10 mm; matrix size = 128 × 128 × 64; voxel size = 0.156 μm × 0.156 μm × 0.156 μm; echo train length (ETL) = 64; and scan time = 2 h 50 m 50 s. During imaging, the respiratory rate of mice was monitored using an MR-compatible physiological monitoring and gating system (SA Instruments Inc., Stony Brook, NY, United States).

### Magnetic resonance spectroscopy

Mice (*n* = 6 for each group) were anesthetized with 1–2% isoflurane, and MR spectroscopy (MRS) was conducted at 21 months of age. Static field homogeneity was adjusted using first- and second-order shims *via* a manual shim. During the experiments, a coil of 72-mm inner diameter for radiofrequency (RF) transmission (Rapid) and a 2-channel array mouse head surface coil (Rapid) for signal reception were used. T2-weighted localizer images were obtained in the coronal axial plane using a multislice TSE protocol (TR/TE_eff_ = 3500/10 ms, echo train length = 6, FOV = 20 mm × 20 mm, slice thickness = 0.8 mm, 2 averages, and image matrix = 128 × 128). Proton ^1^H-MRS of volumes of interest (1.2 × 1.5 × 2.0 mm^3^) centered in the left dorsal hippocampus was performed. Water-suppressed point-resolved spectroscopy (PRESS) pulse sequence was used to measure metabolite levels. The parameters for PRESS data acquisition were as follows: TR/TE = 5000/13.87 ms, number of averages = 384; sweep width = 5 kHz; and number of sampling points = 2048. Outer volume suppression was used, which was interleaved with the water signal suppression using variable-power RF pulses with optimized relaxation delays. A non-water-suppressed reference PRESS spectrum was also acquired (8 averages).

Spectral fitting analysis was performed using LCModel software (version 6.3-1N). Cramer-Rao lower bounds, represented as percentage standard deviations of metabolite estimates (%SD), were obtained from the LCModel to determine the precision of metabolite signal estimates. The experimentally observed spectra of macromolecules and the simulated spectra of the following 12 metabolites were included in the basis set for the LCModel: creatine (Cr), phosphocreatine (PCr), GABA, glucose (Glc), glutamine (Gln), Glu, myo-inositol (mIns), lactate (Lac), *N*-acetylaspartate (NAA), and taurine (Taur). Quantification was performed using the absolute metabolite concentration. LCModel fitting was performed over the spectral range of 1.0–4.4 ppm.

### Image analysis

**FIGURE 1:**
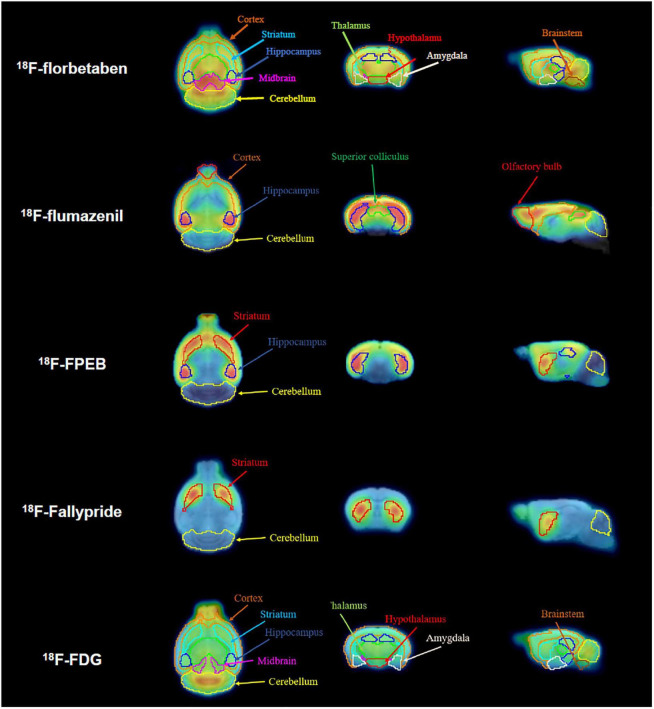
Definition of volumes of interest (VOIs) for all positron emission tomography (PET) tracers. Magnetic resonance (MR) images for the horizontal, coronal, and sagittal planes are presented. Spatially normalized PET images were applied to the VOIs for each radiotracer.

Three-dimensional VOIs were drawn to compare the regional PET uptake in both groups. To this end, dynamic PET images were motion corrected and co-registered with the corresponding magnetic resonance imaging (MRI) for each mouse. The MR images were then spatially normalized to the M. Mirrione T2-weighted mouse brain MRI template found in PMOD software (version 3.4, PMOD Group, Graubünden, Switzerland), and the normalization factor values were applied to the corresponding dynamic PET images. Depending on the characteristics of each PET tracer, VOIs of the target regions were defined on the MRI template (Figure 1). Finally, decay-corrected regional time-activity curves (TACs) of the target regions were obtained. The obtained uptake value, represented as the standardized uptake value (SUV), was determined for each region. SUV values were calculated to normalize the differences in the injected dose and body weight. To compare quantitative values between the groups, we obtained the target-to-reference ratio based on the TACs. We then compared the radioactivity at 30 min, which indicates the beginning of transient equilibrium. Pharmacokinetic parameters such as the area under the curve (AUC) were estimated from the TACs. The AUCs were obtained from 0 to 50 min by the trapezoid rule using the Prism software (version 8, GraphPad Software, Inc., La Jolla, CA, United States).

### Immunohistochemistry

APPswe/PS2 and WT mice were anesthetized using 3% isoflurane in oxygen and perfused with normal saline containing 0.1% heparin (JW Pharmaceutical Corp., Seoul, South Korea) and 4% paraformaldehyde (PFA) solution (Biosesang, South Korea). Mouse brains were removed and post-fixed with formalin (10%). Staining was performed at the Korean Pathology Technical Center (Cheong-ju, South Korea). Briefly, brain samples were embedded in paraffin and cut into 4-μm sections using a microtome (Shandon Finesse ME Microtome, Thermo Fisher Scientific, Waltham, MA, United States). After dewaxing and rehydration, endogenous peroxidase activity was blocked with a peroxidase-blocking solution for 10 min (S2023, DAKO, Copenhagen, Denmark). Brain sections were incubated with mouse anti-6E10 antibody (SIG-39320, diluted 1:1000, Covance, Princeton, NJ, United States) or anti-Aβ_1_-_42_ primary antibody (AB5708P, diluted 1:100, Sigma-Aldrich, Burlington, MA, United States) at 4°C overnight. After washing, the sections were incubated with a secondary antibody (Envision kit, DAKO, Copenhagen, Denmark) at room temperature for 30 min, washed, and incubated at room temperature for 30 min with a rabbit/mouse target retrieval solution (S2369, DAKO, Copenhagen, Denmark). Brain tissues were incubated in 3,3′-diaminobenzidine (DAB) substrate for 3 min (EnVision Detection System, DAKO, Copenhagen, Denmark) and counterstained with Mayer’s hematoxylin (Sigma-Aldrich, Burlington, MA, United States). Digital images of stained sections were obtained using a microscope (CX31, Olympus, Tokyo, Japan). The image was adjusted to a magnification field (100×), and the three areas (100 × 100 μm) were defined as the subregions of the hippocampus (CA1, CA2, and CA3). Aβ_42_ peptides were counted using an image viewing software (Motic VM 3.0, Motic microscopes, Canada).

### Statistical analysis

Quantitative results are expressed as the mean ± SD. All statistical analyses were performed using the GraphPad Prism software (GraphPad Software, Inc., La Jolla, CA, United States). The student’s *t*-test was used to determine statistical significance at a 95% confidence level, and a value of *p* < 0.05 was considered significantly different.

## Results

### Positron emission tomography

#### Glucose positron emission tomography

**FIGURE 2:**
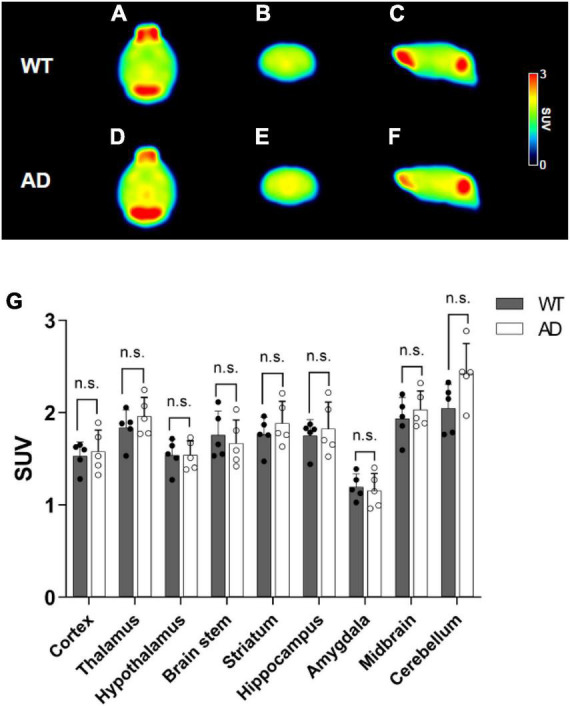
Summed positron emission tomography (PET) images (40–60 min) of ^18^F-FDG in the wild-type (WT) **(A–C)** and Alzheimer’s disease (AD) groups **(D–F)**. In PET images, the columns from left to right show the axial, coronal, and sagittal views. Comparison of the standardized uptake values (SUVs) in terms of the target regions **(G)**. Data are presented as the mean ± SD (*n* = 6).

^18^FDG uptake, a surrogate marker of glucose metabolism, was used to assess neuronal integrity. Lower uptake (hypometabolism) was considered to indicate neurodegeneration. The interpretation of PET images by visual inspection revealed no significant difference in ^18^F-FDG uptake in the target area between the two groups (Figures 2A–F). The mean SUV at 40–60 min for the AD demonstrated no significant difference in all target regions compared to those for the WT group (cortex: WT 1.53 ± 0.15 vs. AD 1.58 ± 0.23, *p* = 0.7278, thalamus: WT 1.84 ± 0.19 vs. AD 1.90 ± 0.22, *p* = 0.6762, hypothalamus: WT 1.54 ± 0.17 vs. AD 1.54 ± 0.16, *p* > 0.9999, brain stem: WT 1.76 ± 0.26 vs. AD 1.67 ± 0.28, *p* = 0.5313, striatum: WT 1.77 ± 0.18 vs. AD 1.80 ± 0.25, *p* = 0.8345, hippocampus: WT 1.75 ± 0.18 vs. AD 1.80 ± 0.31, *p* = 0.7278, amygdala: WT 1.20 ± 0.14 vs. AD 1.16 ± 0.20, *p* = 0.7806, midbrain: WT 1.94 ± 0.23 vs. AD 2.00 ± 0.22, *p* = 0.6762, cerebellum: WT 2.05 ± 0.26 vs. AD 2.13 ± 0.33, *p* = 0.5778, Figure 2G).

#### Amyloid positron emission tomography

**FIGURE 3:**
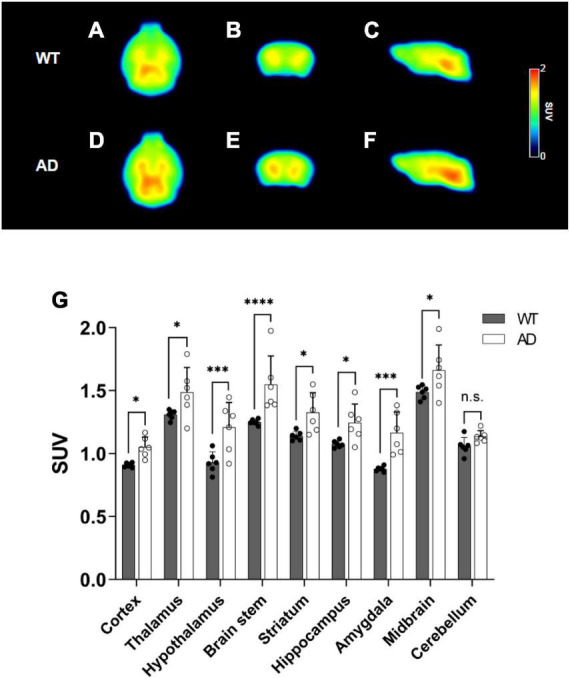
Summed positron emission tomography (PET) images (10–30 min) of ^18^F-florbetaben in the wild-type (WT) **(A–C)** and Alzheimer’s disease (AD) groups **(D–F)**. Comparison of the standardized uptake values (SUVs) for the brain regions **(G)** at 10–30 min. Values are presented as the mean ± SD (*n* = 6). Statistical significance was defined as a *p* value less than 0.05 for comparisons between groups (*p < 0.05, ***p < 0.001 and ****p < 0.0001).

To evaluate the Aβ burden, ^18^F-florbetaben PET scans were performed. Mean PET images showing brain uptake (10–30 min) are depicted in Figures 3A–F, which revealed that radioactivity mainly accumulated in the midbrain, thalamus, and brainstem in the brain. In the TAC group, after approximately 10 min, the AD group exhibited increased ^18^F-florbetaben uptake compared to the WT group (Supplementary Figures 1A–I). The mean SUV at 10–30 min for the hypothalamus, hippocampus, and brain stem of the AD group demonstrated higher uptake than that of the WT (cortex: WT 0.91 ± 0.02 vs. AD 1.05 ± 0.08, *p* = 0.0475, thalamus: WT 1.31 ± 0.04 vs. AD 1.49 ± 0.20, *p* = 0.0142, hypothalamus: WT 0.93 ± 0.08 vs. AD 1.21 ± 0.20, *p* = 0.0002, brain stem: WT 1.25 ± 0.02 vs. AD 1.55 ± 0.23, *p* < 0.0001, striatum: WT 1.14 ± 0.04 vs. AD 1.33 ± 0.16, *p* = 0.0106, hippocampus: WT 1.08 ± 0.03 vs. AD 1.25 ± 0.15, *p* = 0.0193, amygdala: WT 0.88 ± 0.02 vs. AD 1.07 ± 0.16, *p* = 0.0001, midbrain: WT 1.49 ± 0.05 vs. AD 1.66 ± 0.20, *p* = 0.0162, cerebellum: WT 1.06 ± 0.07 vs. AD 1.14 ± 0.05, *p* = 0.2879, Figure 3G). The AUC values of the target regions showed a 2–12% increase, compared with the corresponding values in the WT group (cortex: WT 47.85 ± 0.47 vs. AD 53.55 ± 1.02, *p* < 0.0001, thalamus: WT 67.61 ± 0.80 vs. AD 74.54 ± 1.70, *p* < 0.0001, hypothalamus: WT 47.76 ± 0.92 vs. AD 55.15 ± 2.00, *p* < 0.0001, brain stem: WT 62.99 ± 0.71 vs. AD 69.37 ± 2.94, *p* < 0.0001, striatum: WT 58.59 ± 0.75 vs. AD 62.32 ± 1.91, *p* = 0.0002, hippocampus: WT 56.18 ± 0.63 vs. AD 62.42 ± 1.44, *p* < 0.0001, amygdala: WT 46.35 ± 1.33 vs. AD 50.15 ± 1.84, *p* = 0.0001, midbrain: WT 73.43 ± 0.85 vs. AD 80.71 ± 1.76, *p* < 0.0001, cerebellum: WT 55.78 ± 0.90 vs. AD 61.92 ± 1.98, *p* < 0.0001, Table 1).

**TABLE 1:** Comparison of the regional area under the curve (AUC) values (0–50 min).

Radiotracers	Group	AUCs										
		Cortex	Thalamus	Hypothalamus	Brain stem	Striatum	Hippocampus	Amygdala	Midbrain	Superior colliculus	Olfactory bulb	Cerebellum
^18^F-florbetaben	WT	47.85 ± 0.47	67.61 ± 0.80	47.76 ± 0.92	62.99 ± 0.71	58.59 ± 0.75	56.18 ± 0.63	46.35 ± 1.33	73.43 ± 0.85	–	–	55.78 ± 0.90
	AD	53.55 ± 1.02****	74.54 ± 1.70****	55.15 ± 2.00****	69.37 ± 2.94****	62.32 ± 1.91***	62.42 ± 1.44****	50.15 ± 1.84***	80.71 ± 1.76****	–	–	61.92 ± 1.98****
^18^F-FPEB	WT	–	–	–	–	97.72 ± 4.78	98.32 ± 4.89	–	–	–	–	22.47 ± 0.95
	AD	–	–	–	–	71.65 ± 3.01****	74.64 ± 3.93****	–	–	–	–	21.97 ± 0.48
^18^F-flumazenil	WT	117.4 ± 2.91	–	–	–	–	144.8 ± 9.65	–	–	135.7 ± 9.84	92.86 ± 5.78	76.94 ± 4.58
	AD	134.4 ± 4.75***	–	–	–	–	159.9 ± 6.47***	–	–	171.4 ± 7.79****	111.3 ± 5.93***	78.69 ± 4.03
^18^F-fallypride	WT	–	–	–	–	90.45 ± 2.06	–	–	–	–	–	22.98 ± 1.02
	AD	–	–	–	–	64.88 ± 0.27****	–	–	–	–	–	21.05 ± 0.31

Values are presented as the mean ± SD. Statistical significance was defined as a p value less than 0.05 (***p < 0.001, and ****p < 0.0001 compared to the WT group).

#### Glutamate positron emission tomography

**FIGURE 4:**
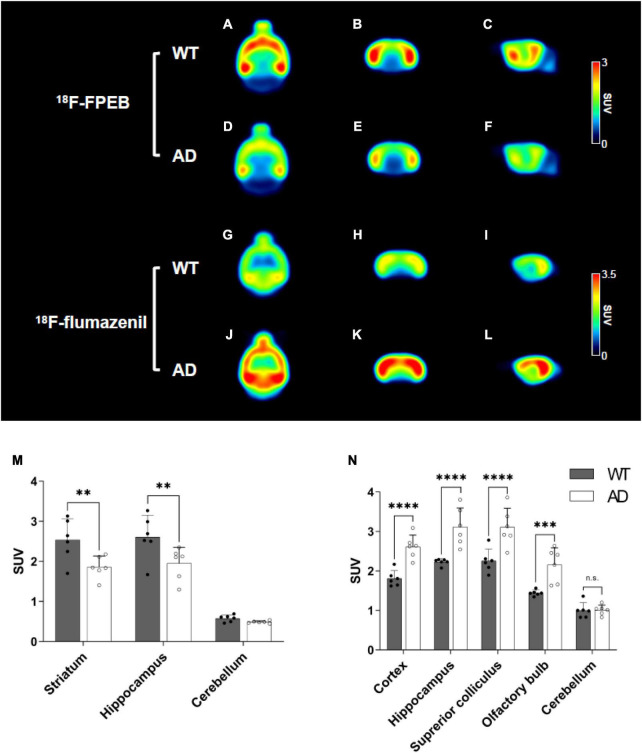
Mean positron emission tomography (PET) images of ^18^F-FPEB [wild-type (WT) **(A–C)** and Alzheimer’s disease (AD) **(D–F)** at 40–60 min] and ^18^F-flumazenil [WT **(G–I)** and AD **(J–L)** at 30–50 min]. Quantification of the radioactivities for the ^18^F-FPEB **(M)** and ^18^F-flumazenil **(N)**. Data are presented as the mean ± SD (*n* = 6). Statistical significance was defined as a *p* value less than 0.05 for comparisons between groups (**p < 0.01, ***p < 0.001, and ****p < 0.0001).

To evaluate the glutamatergic system, we measured the density of the metabotropic glutamate receptor 5 (mGluR5) using ^18^F-FPEB PET. The mean PET images (30–50 min) are shown in Figures 4A–F. Supplementary Figures 2A–C represent the regional TACs. After approximately 10 min, the striatum and hippocampus of the AD group showed lower PET uptake than those of the WT group. The SUVs at 30–50 min of the striatum indicated 26.8% lower uptake in the AD group than in the WT group (WT 2.55 ± 0.52 vs. AD 1.86 ± 0.28 SUV, *p* = 0.0034, Figure 4M). In the hippocampus, the uptake of the AD group was 25.3% lower than that of the WT group (WT 2.61 ± 0.55 vs. AD 1.95 ± 0.40, *p* = 0.0043, Figure 4M). In contrast, the radioactivity in the cerebellum did not differ between the groups (WT 0.58 ± 0.09 SUV vs. AD 0.49 ± 0.03, *p* = 0.6696, Figure 4M). The AUC value of the target region in the AD group showed a 24–27% decrease compared to that of the WT group (striatum: WT 97.72 ± 4.78 vs. AD 71.65 ± 3.01, *p* < 0.0001, hippocampus: WT 98.32 ± 4.89 vs. AD 74.64 ± 3.93, *p* < 0.0001, cerebellum: WT 22.47 ± 0.95 vs. AD 21.97 ± 0.48, *p* = 0.8184, Table 1).

#### GABAergic positron emission tomography

To observe the distribution of GABA_*A*_ receptors, we performed ^18^F-flumazenil PET. A comparative overview of the mean brain ^18^F-flumazenil PET images is presented in Figures 4G–L. The mice in the AD group exhibited higher radioactivity in the target regions (i.e., the cortex, hippocampus, superior colliculus, and olfactory bulb) than that in the WT group. Supplementary Figures 2D–H shows the regional TACs of ^18^F-flumazenil for both groups, which suggests that transient equilibrium was attained at 30 min on average. After approximately 10 min, the mice in the AD group exhibited relatively higher uptake than the WT group. The radioactivity (SUV) of target regions except the cerebellum indicated a 14–35% higher uptake in the AD group than in the WT group 30–50 min (cortex: WT 1.81 ± 0.22 vs. AD 2.61 ± 0.33, *p* = 0.0006, hippocampus: WT 2.23 ± 0.09 vs. AD 3.11 ± 0.54, *p* = 0.0002, superior colliculus: WT 2.26 ± 0.33 vs. AD 3.11 ± 0.53, *p* = 0.0003, olfactory bulb: WT 1.44 ± 0.08 vs. AD 2.16 ± 0.48, *p* = 0.0017, cerebellum: WT 1.00 ± 0.22 vs. AD 1.01 ± 0.14, *p* = 0.9631, Figure 4N). The AD group showed a 10–26% increase in the AUC value of the target regions compared to the WT group (cortex: WT 117.40 ± 2.91 vs. AD 134.40 ± 4.75, *p* = 0.0002, hippocampus: WT 144.80 ± 9.65 vs. AD 159.90 ± 6.47, *p* = 0.0008, superior colliculus: WT 135.70 ± 9.84 vs. AD 171.40 ± 7.79, *p* < 0.0001, olfactory bulb: WT 92.86 ± 5.78 vs. AD 111.30 ± 5.93, *p* < 0.0001, cerebellum: WT 76.94 ± 4.58 vs. AD 78.69 ± 4.03, *p* = 0.6751, Table 1).

#### Dopaminergic positron emission tomography

**FIGURE 5:**
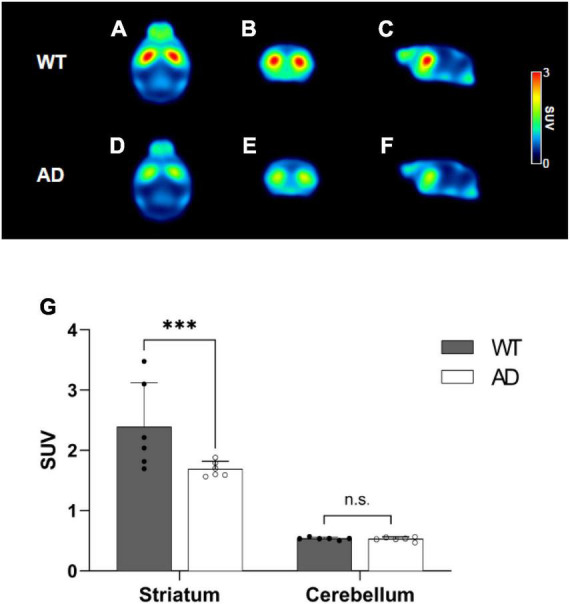
Mean positron emission tomography (PET) images of ^18^F-fallypride for wild-type (WT) **(A–C)** and Alzheimer’s disease (AD) groups **(D–F)**. Comparison of the standardized uptake values (SUVs) for the striatum and cerebellum for ^18^F-fallypride at 30–50 min **(G)**. Data are presented as the mean ± SD (*n* = 6). Statistical significance was defined as a *p* value less than 0.05 for comparisons between groups (****p < 0.0001).

To measure dopamine D2 receptor levels, ^18^F-fallypride PET was performed. Summed PET images showed that striatal uptake was significantly lower in the AD group than in the WT group (Figures 5A–F). In the TAC group, transient equilibrium was reached after an average of 30 min, and the radioactivity of the striatum revealed lower uptake in the AD group than in the WT group (Supplementary Figure 3). The striatal uptake in the AD group at 30–50 min was 29.3% lower than that in the WT group (WT 2.39 ± 0.73 vs. AD 1.69 ± 0.12, *p* = 0.0036, Figure 5G). The AD group exhibited a 28% reduction in striatal AUC values compared to the WT group (WT 90.45 ± 2.06 vs. AD 64.88 ± 0.27, *p* < 0.0001, Table 1).

### Aβ_42_ immunoreactivity

**FIGURE 6:**
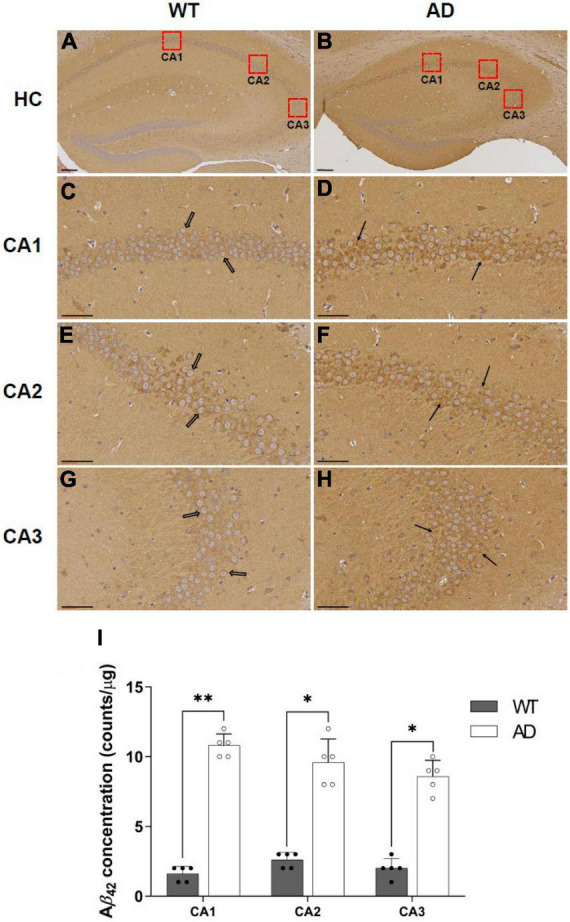
Immunohistochemical staining of Aβ_42_ peptide in the hippocampus for wild-type (WT) and Alzheimer’s disease (AD) mice **(A–H)**. **(A,B)** 100× magnification of the hippocampus, **(C–H)** 400× magnification of the hippocampal regions such as CA1, CA2, and CA3 (Scale bar = 60 μm). The number of Aβ42 peptides in the CA1, CA2, and CA3 regions are expressed as mean ± SD (**I**, *p < 0.05, and **p < 0.005). This analysis was done for all mice (*n* = 5).

To compare Aβ expression levels between AD and WT mice, we analyzed the immunoreactivity of the Aβ_42_ peptide in the hippocampus (Figures 6A–H). The number of positive Aβ_42_ cells in AD mice was significantly higher than that in WT mice. In the CA1 region, amyloid pathology was evident in AD mice; however, it was rarely observed in WT mice (Aβ_42_ peptide: WT 1.60 ± 0.55 vs. AD 10.80 ± 0.84 counts/μm^2^, *p* = 0.0041, Figure 6I). This result was also observed in the CA2 and CA3 regions (CA2: WT 2.60 ± 0.55 vs. AD 9.60 ± 1.67 counts/μm^2^, *p* = 0.0142; CA3: WT 2.00 ± 0.71 vs. AD 8.60 ± 1.14 counts/μm^2^, *p* = 0.0488, Figure 6I). Compared with the Aβ pathology using 6E10 staining, no significant differences were observed between the two groups (Supplementary Figure 4).

### Magnetic resonance spectroscopy

**FIGURE 7:**
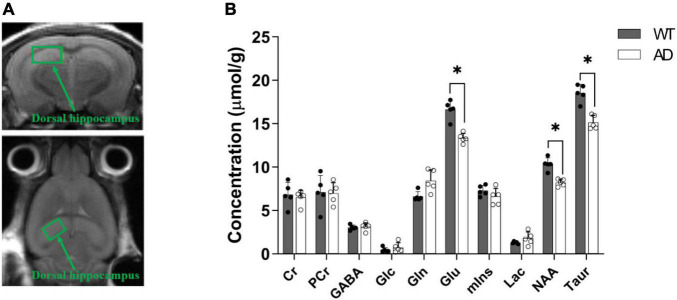
Definition of the volume of interest (i.e., dorsal hippocampus) in magnetic resonance spectroscopy (MRS) **(A)**. Comparison of the neurochemical profiles between the wild-type (WT) and APPswe/PS2 mice **(B)**. Data are presented as the mean ± SD (*n* = 5). A *p*-value < 0.05 was considered statistically significant (*p < 0.05).

To determine the biochemical features of AD, we compared the concentrations of neurochemicals in the dorsal hippocampus of AD and WT mice (Figure 7). Creatine, phosphocreatine, glucose, and myoinositol levels in the AD group were similar to those in WT mice. Although no statistically significant differences were observed, the concentrations of GABA, glutamine, and lactate were higher in the AD group than those in the WT group. However, the AD group exhibited a significant decrease in glutamate (*p* = 0.032), *N*-acetylaspartate (*p* = 0.036), and taurine (*p* = 0.036).

## Discussion

Amyloid pathology induces synaptic dysfunction, neuronal death, and brain shrinkage resulting in various clinical symptoms. Although many researchers have conducted molecular biological studies and developed chemicals to prevent AD progression, no effective therapeutic drug has been developed to date except aducanumab (Aduhelm™). Limited by the scope of molecular imaging, existing studies have evaluated only the effect of a single neurotransmission system in the AD model. However, since many neurotransmission systems are interconnected, a compressive evaluation is required to broaden the understanding of AD pathophysiology. In the present study, we systemically elucidated the effect of Aβ pathology on major neurotransmission systems *in vivo* using functional molecular imaging techniques. First, we confirmed amyloid pathology with PET and observed an imbalance of the excitatory-inhibitory system and a decreased dopaminergic system in APPswe/PS2 mice (21 months of age). Notably, changes in the GABAergic system are prominent among the neurotransmission systems, particularly in the superior colliculus. This implies that the GABAergic system is the most vulnerable to amyloid pathology; therefore, modulation of this system may be an effective target for AD treatment in advanced stage.

The mammalian brain uses glucose as its primary energy source; therefore, glucose metabolism plays a pivotal role in maintaining neuronal function and neurotransmission. The disruption of neuronal glucose metabolism is closely related to synaptic dysfunction (Moon et al., 2014). In patients with AD, reduced cerebral glucose consumption, called “hypometabolism,” is typically observed, indicating neuronal degradation (Costantini et al., 2008). However, several preclinical PET studies have shown findings contradictory to the clinical results of “hypermetabolism” (Luo et al., 2012; Poisnel et al., 2012; Rojas et al., 2013). In the present study, we did not observe any significant differences in ^18^F-FDG uptake between the AD and WT groups. This result is in good agreement with Kuntner’s work. They demonstrated that ^18^F-FDG-PET in Tg2576 mice did not show any significant differences in glucose metabolism in the brain compared with WT mice (Kuntner et al., 2009). These results may be due to the presence of inflammatory cells around amyloid plaques. High astrogliosis and microgliosis can reportedly increase glucose metabolism, which appears to be reflected in the PET images. In the case of APP/PS2 mice, the inflammatory response starts at the age of 9 months and correlates with Aβ deposition (Richards et al., 2003). Further investigation is required to address this issue.

Aβ is regarded as a critical biomarker for AD. In clinical studies, growing evidence suggests that amyloid PET correlates with the pathology of AD (Ikonomovic et al., 2008; Sojkova et al., 2011). However, the suitability of amyloid PET for preclinical use is controversial in the literature. Some studies have successfully monitored the progression of amyloidosis in animal models of AD. Snellman et al. (2013) demonstrated that neocortical ^11^C-PIB uptake is noticeably increased in APP23. Rojas et al. (2013) revealed that 5xFAD mice exhibited a significantly higher uptake of ^18^F-florbetapir and ^11^C-PIB. In contrast, other researchers have observed no difference between the AD and WT groups, despite the pathological confirmation of the deposition of high Aβ. Klunk et al. (2005) also showed no significant uptake of ^11^C-PIB in APP/PS2 mice compared to the WT. Amyloid pathologies may vary depending on the genetically modified mouse. To address this issue, Brendel et al. (2015) performed a comparative amyloid PET and cross-sectional histological study to identify suitable transgenic mice with APPswe, APPswe/PS2, APPswe/PS1G384A, and APPswe/PS1dE9. They suggested that APPswe/PS2 transgenic mice had the highest brain and progressive radioactivity uptake with age (Brendel et al., 2015). Therefore, we selected APPswe/PS2 mice for the present study and observed higher brain uptake values in these transgenic mice than in wild-type mice.

Glutamatergic and GABAergic neurons are the two major types of synapses in the central nervous system (CNS), which play a role in achieving a coordinated balance between excitation and inhibition events, as well as learning and memory (Mathew et al., 2005; Foster and Kemp, 2006). Li et al. (2016) noted that both neurotransmission systems might undergo pathological changes in AD. In this study, we evaluated the *in vivo* changes in mGluR5 in APPswe/PS2 mice and observed that the uptake of ^18^F-FPEB in the AD group was considerably lower than that in the WT group, which is consistent with our previous work and a clinical study conducted by Mecca et al. (2020) (Lee et al., 2018). This reduced expression of mGluR5 indicated the diminished availability of binding sites for radiotracers since mGluR5 is a scaffold for Aβ oligomers and cellular prion proteins (Um et al., 2013).

γ-aminobutyric acid is the primary inhibitory neurotransmitter that binds to three types of receptors (GABA_A,_ GABA_B_, and GABA_C_) (Barnard et al., 1998). As GABA_A_ receptors are widely expressed in the CNS, they mediate the inhibition of most brain functions (Kwakowsky et al., 2018). A previous study reported that abnormal GABA-mediated signaling plays a vital role in neurological diseases, including AD (Rudolph and Möhler, 2014). Notably, we observed significantly higher GABAergic PET uptake in specific brain regions in the AD group than that in the WT group. These results indicate that the inhibitory neurotransmitter systems were activated in the AD group. This finding contradicts those of previous studies. This may be due to differences in the timing of the experiments or the pathological stage. Rissman et al. (2004) reported that a decrease in the expression of GABA_A_ receptors is an early event in patients with AD. Pascual et al. (2012) also demonstrated that patients with early-stage AD showed decreased GABA PET. The distinct mechanism for the increase in GABA-PET uptake in AD is unknown; however, this result may be due to the intervention of GABA_A_ receptors. In the mammalian brain, the GABA_A_ receptor contains two α subunits, two β subunits, and one γ subunit (Olsen and Sieghart, 2008). Altered GABA_*A*_ receptor subunits are observed in AD and significantly impact neuronal function and disease progression (Kwakowsky et al., 2018). Iwakiri et al. (2009) reported that the γ-subunit of hippocampal GABA_A_ receptors is upregulated in patients with AD in the immunohistochemical findings. In addition, Limon et al. (2012) demonstrated that the α2, β1, and γ1 subunits in patients with AD are upregulated at the mRNA and protein levels. ^18^F-flumazenil specifically binds to the binding sites between α and γ of the GABA_A_ receptor; therefore, it seems to increase GABA PET uptake (Palner et al., 2016). A possible explanation is that AD pathology is characterized by the upregulation of the GABAergic system by reactive astrocytes. Under pathological conditions, reactive astrocytes release GABA, which eventually results in overactivation of the GABA_A_ receptor. Jo et al. (2014) reported that reactive astrocytes produced by Aβ generate abnormally upregulated GABA in an APP/PS1 mouse model and participate in AD pathology. Wu et al. observed high levels of GABA in reactive astrocytes in the hippocampus of 5xFAD mice compared with that in WT (Wu et al., 2014) to evaluate changes in the dynamics of the GABAergic system in pathological stages.

Dopamine is another major neurotransmitter found in the CNS. In AD, striatal dopamine levels are known to be associated with cognitive impairments, suggesting the potential of dopaminergic neurons as a treatment target for AD (Kumar and Patel, 2007). However, the pathological mechanism of dopamine in AD is not fully understood. In the present study, we observed significantly lower brain uptake of ^18^F-fallypride in the AD group. This implies that alterations in striatal dopamine D2 receptors are part of the pathological profile of AD (Pizzolato et al., 1996). Previous studies have supported our results. Immunocytochemistry studies reported by Kumar and Patel (2007) revealed that D_2_R expression was moderately reduced in the frontal cortex of the AD group compared with that in the WT group. Pizzolato et al. (1996) reported that striatal dopamine D2 receptors were reduced in patients with AD compared with those in the control group. Kemppainen et al. (2003) also demonstrated that dopaminergic PET uptake decreased in the hippocampal and temporal cortical regions of patients with AD. The ventral tegmental area (VTA) is a group of neurons located in the midbrain that sends dopaminergic projections to both the limbic and cortical areas. The VTA contains dopaminergic neurons (65%), GABAergic neurons (30%), and glutamatergic neurons (approximately 5%) (Swanson, 1982; Margolis et al., 2006; Nair-Roberts et al., 2008; Yamaguchi et al., 2011). Previous studies have demonstrated that dopaminergic neurons are inversely correlated with GABA neurons *in vivo* (Zhou et al., 2009). In this study, we also observed activation of the GABAergic system and simultaneous suppression of the dopaminergic system in an animal model of AD.

In MRS experiments, no difference in creatine and phosphocreatine levels indicated a similar cellular energy metabolism between the AD and WT groups (Ford and Crewther, 2016). This may be linked to glucose levels. A low glutamate + glutamine (Glx) concentration in AD compared with that in the WT indicates decreased glutamatergic neurotransmission. Antuono et al. (2001) also reported reduced Glx levels in patients with AD. Glutamate and *N*-acetylaspartate are related to neuronal integrity and the number of living neurons, respectively (Ramadan et al., 2013; Glodzik et al., 2015). Taurine levels are key indicators of neurogenesis (Kulak et al., 2010). Therefore, our MRS data imply that AD mice have damaged neuronal integrity, a reduced number of neurons, and neurogenesis. These results were also consistent with those of our previous work using 5xFAD mice. The levels of glutamate, *N*-acetylaspartate, and taurine in the AD group were significantly lower than those in the WT group at 5 months of age (Lee et al., 2019). Lin et al. (2014) also reported that AD mice displayed significantly lower glutamate and *N*-acetylaspartate compared to that in the control group. Patients with AD also showed a decreased *N*-acetylaspartate concentration compared to that in healthy individuals (Dixon et al., 2002; Kantarci et al., 2007).

The most crucial consideration in applying the five different types of PET studies to clinical patients is the severity of the disease and radiation exposure. First, although the specific role of the individual neurotransmission system in the progression of AD has not yet been clarified, the sensitivity of the neurotransmission system may vary depending on the severity of AD. Second, in terms of clinical research, there are practical restrictions on acquiring five different types of PET scans from patients. According to the Radioactive Drug Research Committee guidelines in the United States, the effective dose for the whole body should not exceed 30 or 50 mSv/year (FDA 21 CFR 361.1). Previous studies have reported that the whole-body effect dose of PET scans used in the present study was 0.015–0.021 mSv/MBq (mean 0.018 mSv/MBq, Van Laere et al., 2010; Kessler et al., 2014; Sabri et al., 2015; Srinivasan et al., 2020). For an injected dose of 370 MBq, the whole-body was 6.68 mSv/each and 33.4 mSv/five study. This value does not exceed the annual permissible dose; however, it can be challenging to perform follow-up or additional radiation exposure studies within a year. To conduct multiple PET studies with a cycle of over a year, two or three PET scans may be conducted each year.

The significance of the present study is that it is possible to identify the role of specific neurotransmission systems in elucidating the pathophysiology of AD by conducting multiple PET studies, which are difficult to apply in clinical practice. We plan to conduct longitudinal studies to determine the specific role of a certain neurotransmission system in AD progression.

A limitation of the present study is the absence of behavioral studies. Hence, we are uncertain whether a specific neurotransmission system affects the clinical symptoms, including cognitive function and mood-related changes.

## Conclusion

Overall, we determined amyloid pathology by increased brain uptake of amyloid PET, which was validated by immunohistochemistry. This pathology induces an imbalance in the excitatory-inhibitory neurotransmission system and a reduction in the dopaminergic system. AD mice exhibited lower concentrations of major neurochemicals, such as Glu, NAA, and Taur. In general, our results demonstrate that AD pathology causes the deterioration of neurotransmission systems. This expands the understanding of the pathophysiology of AD and may help develop appropriate treatment strategies for AD.

## Data availability statement

The original contributions presented in this study are included in the article/Supplementary material, further inquiries can be directed to the corresponding author.

## Ethics statement

The animal study was reviewed and approved by the Institutional Animal Care and Use Committee of the Korea Institute of Radiological and Medical Sciences (KIRAMS).

## Author contributions

SO and JC: conceptualization, data curation, writing—original manuscript, review, and editing. JC and YL: funding acquisition. NL: investigation. KN, KK, and KL: methodology. All authors have read and agreed to the published version of the manuscript.

## References

[B1] Antuono P. G. Jones J. L. Wang Y. Li S. J. (2001). Decreased glutamate + glutamine in Alzheimer’s disease detected in vivo with ^1^H-MRS at 0.5 T. *Neurology* 56 737–742. 10.1212/WNL.56.6.737 11274307

[B2] Barnard E. A. Skolnick P. Olsen R. W. Mohler H. Sieghart W. Biggio G. (1998). International Union of Pharmacology. XV. Subtypes of γ-aminobutyric acidA receptors: Classification on the basis of subunit composition, pharmacology, and function. *Pharmacol. Rev.* 50 291–313. 9647870

[B3] Brendel M. Jaworska A. Grießinger E. Rötzer C. Burgold S. Gildehaus F. J. (2015). Cross-sectional comparison of small animal [^18^F]-florbetaben amyloid-PET between transgenic AD mouse models. *PLoS One* 10:e0116678. 10.1371/journal.pone.0116678 25706990 PMC4338066

[B4] Costantini L. C. Barr L. J. Vogel J. L. Henderson S. T. (2008). Hypometabolism as a therapeutic target in Alzheimer’s disease. *BMC Neurosci.* 9 (Suppl. 2):S16. 10.1186/1471-2202-9-S2-S16 19090989 PMC2604900

[B5] Dixon R. M. Bradley K. M. Budge M. M. Styles P. Smith A. D. (2002). Longitudinal quantitative proton magnetic resonance spectroscopy of the hippocampus in Alzheimer’s disease. *Brain* 125 2332–2341. 10.1093/brain/awf226 12244089

[B6] Ford T. C. Crewther D. P. (2016). A comprehensive review of the 1H-MRS metabolite spectrum in autism spectrum disorder. *Front. Mol. Neurosci.* 9:14. 10.3389/fnmol.2016.00014 27013964 PMC4783404

[B7] Foster A. C. Kemp J. A. (2006). Glutamate- and GABA-based CNS therapeutics. *Curr. Opin. Pharmacol.* 6 7–17. 10.1016/j.coph.2005.11.005 16377242

[B8] Glodzik L. Sollberger M. Gass A. Gokhale A. Rusinek H. Babb J. S. (2015). Global N-acetylaspartate in normal subjects, mild cognitive impairment and Alzheimer’s disease patients. *J. Alzheimers Dis.* 43 939–947. 10.3233/JAD-140609 25125458 PMC4445651

[B9] Ikonomovic M. D. Klunk W. E. Abrahamson E. E. Mathis C. A. Price J. C. Tsopelas N. D. (2008). Post-mortem correlates of in vivo PiB-PET amyloid imaging in a typical case of Alzheimer’s disease. *Brain* 131 1630–1645. 10.1093/brain/awn016 18339640 PMC2408940

[B10] Iwakiri M. Mizukami K. Ikonomovic M. D. Ishikawa M. Abrahamson E. E. DeKosky S. T. (2009). An immunohistochemical study of GABA_A_ receptor gamma subunits in Alzheimer’s disease hippocampus: Relationship to neurofibrillary tangle progression. *Neuropathology* 29 263–269. 10.1111/j.1440-1789.2008.00978.x 19019179 PMC3078755

[B11] Jo S. Yarishkin O. Hwang Y. J. Chun Y. E. Park M. Woo D. H. (2014). GABA from reactive astrocytes impairs memory in mouse models of Alzheimer’s disease. *Nat. Med.* 20 886–896. 10.1038/nm.3639 24973918 PMC8385452

[B12] Kang K. J. Oh S. J. Nam K. R. Ahn H. Park J. Lee K. C. (2021). Validation of image qualities of a novel four-mice bed PET system as an oncological and neurological analysis tool. *J. Imaging* 7:43. 10.3390/jimaging7030043 34460699 PMC8321312

[B13] Kantarci K. Weigand S. D. Petersen R. C. Boeve B. F. Knopman D. S. Gunter J. (2007). Longitudinal ^1^H MRS changes in mild cognitive impairment and Alzheimer’s disease. *Neurobiol. Aging* 28 1330–1339. 10.1016/j.neurobiolaging.2006.06.018 16860440 PMC2766807

[B14] Kas A. Migliaccio R. Tavitian B. (2020). A future for PET imaging in Alzheimer’s disease. *Eur. J. Nucl. Med. Mol. Imaging* 47 231–234. 10.1007/s00259-019-04640-w 31858177

[B15] Kemppainen N. Laine M. Laakso M. P. Kaasinen V. Någren K. Vahlberg T. (2003). Hippocampal dopamine D2 receptors correlate with memory functions in Alzheimer’s disease. *Eur. J. Neurosci.* 18 149–154. 10.1046/j.1460-9568.2003.02716.x 12859348

[B16] Kessler R. M. Seibyl J. Cowan R. L. Zald D. Young J. S. Ansari M. S. (2014). Radiation dosimetry of18F-FPEB in humans. *J. Nuclear Med.* 55 1119–1121. 10.2967/jnumed.113.133843 24799618

[B17] Klunk W. E. Lopresti B. J. Ikonomovic M. D. Lefterov I. M. Koldamova R. P. Abrahamson E. E. (2005). Binding of the positron emission tomography tracer Pittsburgh Compound-B reflects the amount of amyloid-β in Alzheimer’s Disease brain but not in transgenic mouse brain. *J. Neurosci.* 25 10598–10606. 10.1523/JNEUROSCI.2990-05.2005 16291932 PMC6725842

[B18] Kulak A. Duarte J. M. N. Do K. Q. Gruetter R. (2010). Neurochemical profile of the developing mouse cortex determined by in vivo ^1^H NMR spectroscopy at 14.1T and the effect of recurrent anaesthesia. *J. Neurochem.* 115 1466–1477. 10.1111/j.1471-4159.2010.07051.x 20946416

[B19] Kumar U. Patel S. C. (2007). Immunohistochemical localization of dopamine receptor subtypes (D_1_R-D_5_R) in Alzheimer’s disease brain. *Brain Res.* 1131 187–196. 10.1016/j.brainres.2006.10.049 17182012

[B20] Kuntner C. Kesner A. L. Bauer M. Kremslehner R. Wanek T. Mandler M. (2009). Limitations of small animal PET imaging with [^18^F]FDDNP and FDG for quantitative studies in a transgenic mouse model of Alzheimer’s disease. *Mol. Imaging Biol.* 11 236–240. 10.1007/s11307-009-0198-z 19214638

[B21] Kwakowsky A. Calvo-Flores Guzmán B. Pandya M. Turner C. Waldvogel H. J. Faull R. L. (2018). GABA_A_ receptor subunit expression changes in the human Alzheimer’s disease hippocampus, subiculum, entorhinal cortex and superior temporal gyrus. *J. Neurochem.* 145 374–392. 10.1111/jnc.14325 29485232

[B22] Lee M. Lee H. J. Jeong Y. J. Oh S. J. Kang K. J. Han S. J. (2019). Age dependency of mGluR5 availability in 5xFAD mice measured by PET. *Neurobiol. Aging* 84 208–216. 10.1016/j.neurobiolaging.2019.08.006 31570178

[B23] Lee M. Lee H. J. Park I. S. Park J. A. Kwon Y. J. Ryu Y. H. (2018). Aβ pathology downregulates brain mGluR5 density in a mouse model of Alzheimer. *Neuropharmacology* 133 512–517. 10.1016/j.neuropharm.2018.02.003 29427650

[B24] Li Y. Sun H. Chen Z. Xu H. Bu G. Zheng H. (2016). Implications of GABAergic neurotransmission in Alzheimer’s disease. *Front. Aging Neurosci.* 23:31. 10.3389/fnagi.2016.00031 26941642 PMC4763334

[B25] Limon A. Reyes-Ruiz J. M. Miledi R. (2012). Loss of functional GABA_*A*_ receptors in the Alzheimer diseased brain. *Proc. Natl. Acad. Sci. U.S.A.* 109 10071–10076. 10.1073/pnas.1204606109 22691495 PMC3382476

[B26] Lin Y. Yao J. Chen Y. Pang L. Li H. Cao Z. (2014). Hippocampal neurochemical changes in senescent mice induced with chronic injection of D-Galactose and NaNO_2_: An in vitro high-resolution NMR spectroscopy study at 9.4T. *PLoS One* 9:e88562. 10.1371/journal.pone.0088562 24533108 PMC3922890

[B27] Lu F. Yuan Z. (2015). PET/SPECT molecular imaging in clinical neuroscience: Recent advances in the investigation of CNS diseases. *Quant. Imaging Med. Surg.* 5 433–447. 10.3978/j.issn.2223-4292.2015.03.16 26029646 PMC4426104

[B28] Luo F. Rustay N. R. Ebert U. Hradil V. P. Cole T. B. Llano D. A. (2012). Characterization of 7- and 19-month-old Tg2576 mice using multimodal in vivo imaging: Limitations as a translatable model of Alzheimer’s disease. *Neurobiol. Aging* 33 933–944. 10.1016/j.neurobiolaging.2010.08.005 20961663

[B29] Margolis E. B. Lock H. Hjelmstad G. O. Fields H. L. (2006). The ventral tegmental area revisited: Is there an electrophysiological marker for dopaminergic neurons? *J. Physiol.* 577 907–924. 10.1113/jphysiol.2006.117069 16959856 PMC1890372

[B30] Mathew S. J. Keegan K. Smith L. (2005). Glutamate modulators as novel interventions for mood disorders. *Braz. J. Psychiatry* 27 243–248. 10.1590/s1516-44462005000300016 16224615

[B31] Mecca A. P. McDonald J. W. Michalak H. R. Godek T. A. Harris J. E. Pugh E. A. (2020). PET imaging of mGluR5 in Alzheimer’s disease. *Alzheimers. Res. Ther.* 12:15. 10.1186/s13195-020-0582-0 31954399 PMC6969979

[B32] Moon B. S. Park J. H. Lee H. J. Lee B. C. Kim S. E. (2014). Routine production of [^18^F]flumazenil from iodonium tosylate using a sample pretreatment method: A 2.5-year production report. *Mol. Imaging Biol.* 16 619–625. 10.1007/s11307-014-0738-z 24788440

[B33] Mosconi L. (2013). Glucose metabolism in normal aging and Alzheimer’s disease: Methodological and physiological considerations for PET studies. *Clin. Transl. Imaging* 1 10.1007/s40336-013-0026-y. 10.1007/s40336-013-0026-y 24409422 PMC3881550

[B34] Nair-Roberts R. G. Chatelain-Badie S. D. Benson E. White-Cooper H. Bolam J. P. Ungless M. A. (2008). Stereological estimates of dopaminergic, GABAergic and glutamatergic neurons in the ventral tegmental area, substantia nigra and retrorubral field in the rat. *Neuroscience* 152 1024–1031. 10.1016/j.neuroscience.2008.01.046 18355970 PMC2575227

[B35] Olsen R. W. Sieghart W. (2008). International union of pharmacology. LXX. Subtypes of γ-aminobutyric acid(A) receptors: Classification on the basis of subunit composition, pharmacology, and function. Update. *Pharmacol. Rev*. 60, 243–260. 10.1124/pr.108.00505 18790874 PMC2847512

[B36] Palner M. Beinat C. Banister S. Zanderigo F. Park J. H. Shen B. (2016). Effects of common anesthetic agents on [^18^F]flumazenil binding to the GABAA receptor. *EJNMMI Res.* 6:80. 10.1186/s13550-016-0235-2 27826950 PMC5101239

[B37] Pan X. Kaminga A. C. Wen S. W. Wu X. Acheampong K. Liu A. (2019). Dopamine and dopamine receptors in Alzheimer’s disease: A systematic review and network meta-analysis. *Front. Aging Neurosci.* 11:175. 10.3389/fnagi.2019.00175 31354471 PMC6637734

[B38] Pascual B. Prieto E. Arbizu J. Marti-Climent J. M. Peñuelas I. Quincoces G. (2012). Decreased carbon-11-flumazenil binding in early Alzheimer’s disease. *Brain* 135 2817–2825. 10.1093/brain/aws210 22961552

[B39] Pizzolato G. Chierichetti F. Fabbri M. Cagnin A. Dam M. Ferlin G. (1996). Reduced striatal dopamine receptors in Alzheimer’s disease: Single photon emission tomography study with the D2 tracer [^123^I]-IBZM. *Neurology* 47 1065–1068. 10.1212/wnl.47.4.1065 8857746

[B40] Poisnel G. Hérard A.-S. El Tannir El Tayara N. Bourrin E. Volk A. Kober F. (2012). Increased regional cerebral glucose uptake in an APP/PS1 model of Alzheimer’s disease. *Neurobiol. Aging* 33 1995–2005. 10.1016/j.neurobiolaging.2011.09.026 22079157 PMC3666917

[B41] Ramadan S. Lin A. Stanwell P. (2013). Glutamate and glutamine: A review of in vivo MRS in the human brain. *NMR Biomed.* 26 1630–1646. 10.1002/nbm.3045 24123328 PMC3849600

[B42] Richards J. G. Higgins G. A. Ouagazzal A. Ozmen L. Kew J. N. C. Bohrmann B. (2003). PS2APP transgenic mice, coexpressing hPS2mut and hAPPswe, show age-related cognitive deficits associated with discrete brain amyloid deposition and inflammation. *J. Neurosci.* 23 8989–9003. 10.1523/JNEUROSCI.23-26-08989.2003 14523101 PMC6740398

[B43] Rissman R. A. Bennett D. A. Armstrong D. M. (2004). Subregional analysis of GABA_A_ receptor subunit mRNAs in the hippocampus of older persons with and without cognitive impairment. *J. Chem. Neuroanat.* 28 17–25. 10.1016/j.jchemneu.2004.05.003 15363487

[B44] Rojas S. Herance J. R. Gispert J. D. Abad S. Torrent É Jiménez X. (2013). In vivo evaluation of amyloid deposition and brain glucose metabolism of 5XFAD mice using positron emission tomography. *Neurobiol. Aging* 34 1790–1798. 10.1016/j.neurobiolaging.2012.12.027 23402900

[B45] Rowley P. A. Samsonov A. A. Betthauser T. J. Pirasteh A. Johnson S. C. Eisenmenger L. B. (2020). Amyloid and Tau PET imaging of alzheimer disease and other neurodegenerative conditions. *Semin. Ultrasound CT MR* 41 572–583. 10.1053/j.sult.2020.08.011 33308496

[B46] Rudolph U. Möhler H. (2014). GABA_A_ receptor subtypes: Therapeutic potential in down syndrome, affective disorders, schizophrenia, and autism. *Chang. Rev. Pharmacol. Toxicol.* 54 483–507. 10.1146/annurev-pharmtox-011613-135947 24160694 PMC3997216

[B47] Sabri O. Seibyl J. Rowe C. Barthel H. (2015). Beta-amyloid imaging with florbetaben. *Clin. Transl. Imaging* 3 13–26. 10.1007/s40336-015-0102-6 25741488 PMC4339690

[B48] Sarawagi A. Soni N. D. Patel A. B. (2021). Glutamate and GABA homeostasis and neurometabolism in major depressive disorder. *Front. Psychiatry.* 27:637863. 10.3389/fpsyt.2021.637863 33986699 PMC8110820

[B49] Seok Moon B. Hyung Park J. Jin Lee H. Sun Kim J. Sup Kil H. Se Lee B. (2010). Highly efficient production of [^18^F]fallypride using small amounts of base concentration. *Appl. Radiat. Isot.* 68 2279–2284. 10.1016/j.apradiso.2010.06.016 20609592

[B50] Serrano-Pozo A. Frosch M. P. Masliah E. Hyman B. T. (2011). Neuropathological alterations in Alzheimer disease. *Cold Spring Harb. Perspect. Med.* 1:a006189. 10.1101/cshperspect.a006189 22229116 PMC3234452

[B51] Snellman A. López-Picón F. R. Rokka J. Salmona M. Forloni G. Scheinin M. (2013). Longitudinal amyloid imaging in mouse brain with ^11^C-PIB: Comparison of APP23, Tg2576, and APPswe-PS1dE9 mouse models of Alzheimer disease. *J. Nucl. Med.* 54 1434–1441. 10.2967/jnumed.112.110163 23833271

[B52] Sojkova J. Driscoll I. Iacono D. Zhou Y. Codispoti K. E. Kraut M. A. (2011). In vivo Fibrillar β-amyloid detected using [^11^C]PiB positron emission tomography and neuropathologic assessment in older adults. *Arch. Neurol.* 68 232–240. 10.1001/archneurol.2010.357 21320990 PMC3082956

[B53] Srinivasan S. Crandall J. P. Gajwani P. Sgouros G. Mena E. Lodge M. A. (2020). Human radiation dosimetry for orally and intravenously administered ^18^F-FDG. *J. Nuclear Med.* 61 613–619. 10.2967/jnumed.119.233288 31628217 PMC9374043

[B54] Swanson L. W. (1982). The projections of the ventral tegmental area and adjacent regions: A combined fluorescent retrograde tracer and immunofluorescence study in the rat. *Brain Res. Bull.* 9 321–353. 10.1016/0361-9230(82)90145-9 6816390

[B55] Tarawneh R. Holtzman D. M. (2012). The clinical problem of symptomatic Alzheimer disease and mild cognitive impairment. *Cold Spring Harb. Perspect. Med.* 2:a006148. 10.1101/cshperspect.a006148 22553492 PMC3331682

[B56] Um J. W. Kaufman A. C. Kostylev M. Heiss J. K. Stagi M. Takahashi H. (2013). Metabotropic glutamate receptor 5 is a Co-Receptor for Alzheimer Aβ oligomer bound to cellular prion protein. *Neuron* 79 887–902. 10.1016/j.neuron.2013.06.036 24012003 PMC3768018

[B57] Van Laere K. Varrone A. Booij J. Vander Borght T. Nobili F. Kapucu ÖL. (2010). EANM procedure guidelines for brain neurotransmission SPECT/PET using dopamine D2 receptor ligands, version 2. *Eur. J. Nuclear Med. Mol. Imaging* 37 434–442. 10.1007/s00259-009-1265-z 19838704

[B58] WHO Guidelines (2019). *Risk reduction of cognitive decline and dementia. World Health Organization. Licence: CC BYNC-SA 3.0 IGO*. Geneva: World Health Organization. 31219687

[B59] Wu Z. Guo Z. Gearing M. Chen G. (2014). Tonic inhibition in dentate gyrus impairs long-term potentiation and memory in an Alzhiemer’ s disease model. *Nat. Commun.* 5:4159. 10.1038/ncomms5159 24923909 PMC4159602

[B60] Yamaguchi T. Wang H. L. Li X. Ng T. H. Morales M. (2011). Mesocorticolimbic glutamatergic pathway. *J. Neurosci.* 31 8476–8490. 10.1523/JNEUROSCI.1598-11.2011 21653852 PMC6623324

[B61] Zhou F. W. Jin Y. Matta S. G. Xu M. Zhou F. M. (2009). An ultra-short dopamine pathway regulates basal ganglia output. *J. Neurosci.* 29 10424–10435. 19692618 10.1523/JNEUROSCI.4402-08.2009 PMC3596265

