# Corrigendum: Amyloid pathology induces dysfunction of systemic neurotransmission in aged APPswe/PS2 mice

**DOI:** 10.3389/fnins.2022.1014128

**Published:** 2022-10-17

**Authors:** Se Jong Oh, Namhun Lee, Kyung Rok Nam, Kyung Jun Kang, Sang Jin Han, Kyo Chul Lee, Yong Jin Lee, Jae Yong Choi

**Affiliations:** ^1^Division of Applied RI, Korea Institute of Radiological and Medical Sciences, Seoul, South Korea; ^2^Radiological and Medico-Oncological Sciences, University of Science and Technology (UST), Seoul, South Korea

**Keywords:** positron emission tomography, Alzheimer's disease, APPswe/PS2, beta amyloid, neurotransmitter

In the published article, there was an error in Figure 1 as published. The term of the fourth row of the Figure 1 image was displayed as “^18^F-FP-CIT.” The correct term is “^18^F-Fallypride.” The corrected Figure 1 appears below.

**Figure 1:**
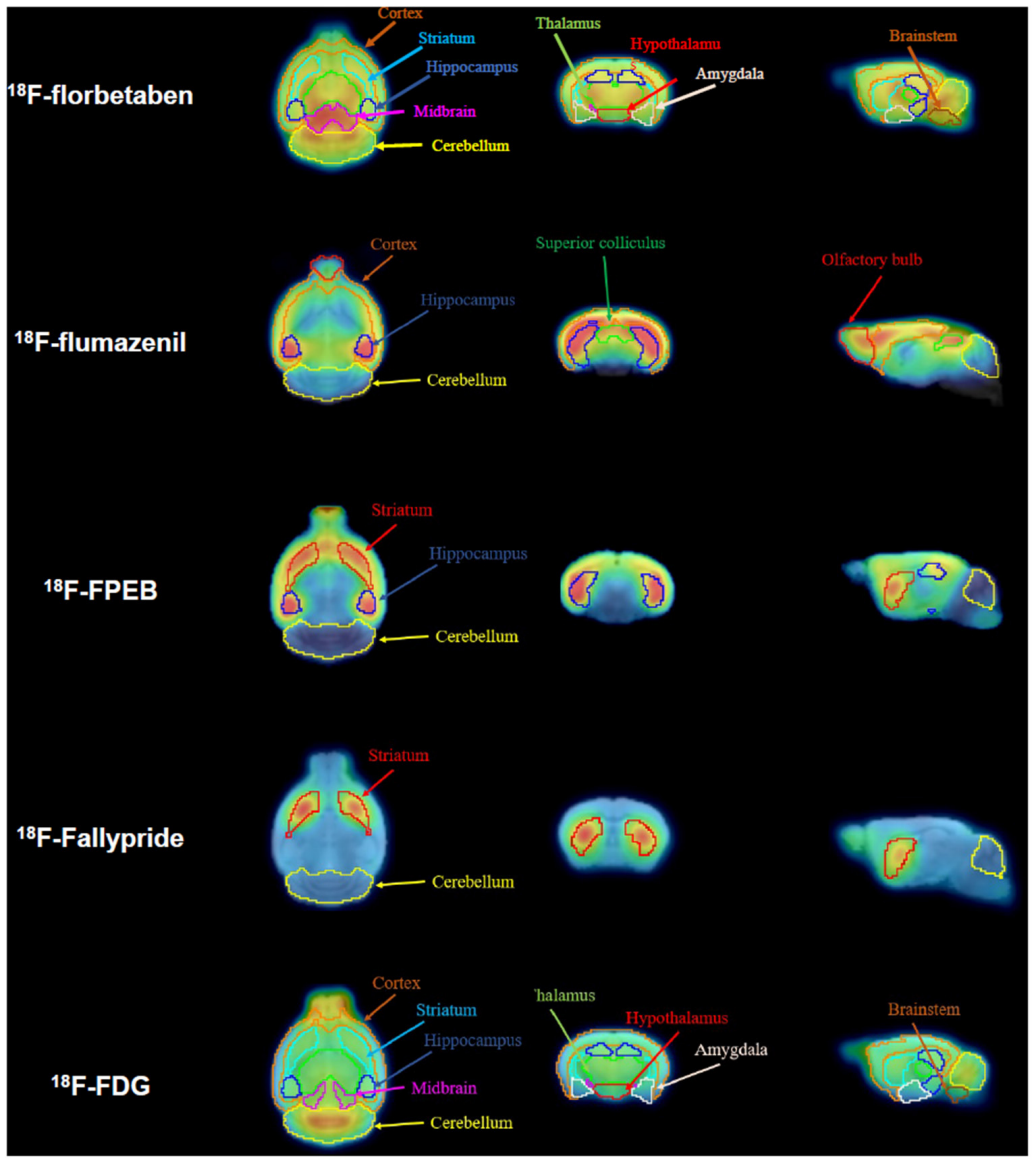
Definition of volumes of interest (VOIs) for all positron emission tomography (PET) tracers. Magnetic resonance (MR) images for the horizontal, coronal, and sagittal planes are presented. Spatially normalized PET images were applied to the VOIs for each radiotracer.

The authors apologize for this error and state that this does not change the scientific conclusions of the article in any way. The original article has been updated.

## Publisher's note

All claims expressed in this article are solely those of the authors and do not necessarily represent those of their affiliated organizations, or those of the publisher, the editors and the reviewers. Any product that may be evaluated in this article, or claim that may be made by its manufacturer, is not guaranteed or endorsed by the publisher.

