# Supplementary material for: Amyloid pathology induces dysfunction of systemic neurotransmission in aged APPswe/PS2 mice
Source: Front Neurosci. 2022 Aug 5;16:930613. doi: 10.3389/fnins.2022.930613 (PMC9389227; doi:10.3389/fnins.2022.930613)
Supplement: Supplementary file 1 [file Data_Sheet_1.docx]

**Amyloid pathology induces dysfunction of systemic neurotransmission in aged APPswe/PS2 mice**

**Se Jong Oh^1^, Namhun Lee^1^, Kyung Rok Nam^1^, Kyung Jun Kang^1^, Sang Jin Han^1^, Kyo Chul Lee^1^, Yong Jin Lee^1^, Jae Yong Choi^1,2*^**

^1^Division of Applied RI, Korea Institute of Radiological and Medical Sciences, Seoul, Korea

^2^Radiological and Medico-Oncological Sciences, University of Science and Technology (UST), Seoul, Korea

*** Correspondence:**Jae Yong Choi
[smhany@kirams.re.kr](mailto:smhany@kirams.re.kr)


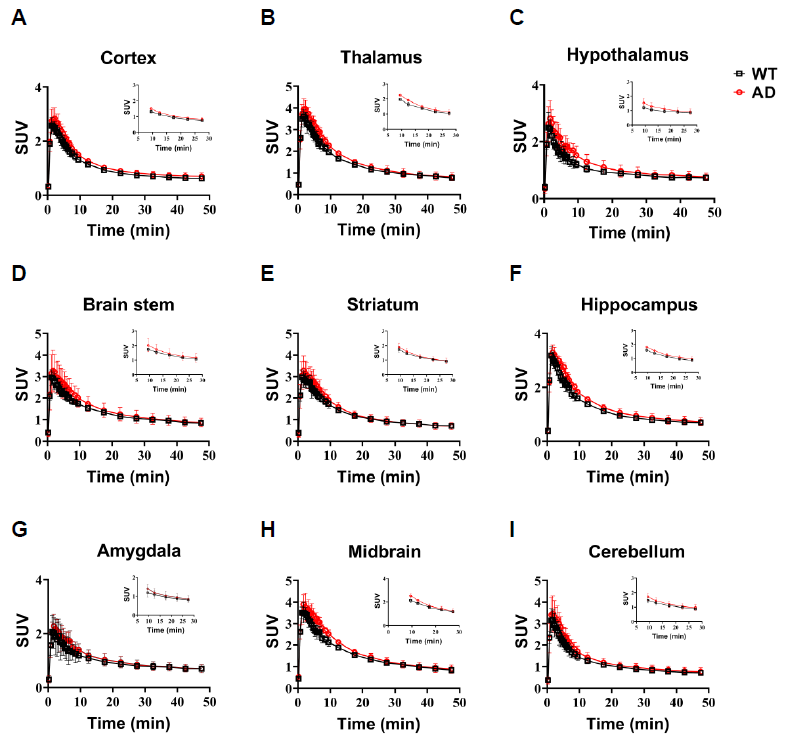


Figure S1. Time-activity curves of ^18^F-florbetaben for the cortex (A), thalamus (B), hypothalamus (C), brain stem (D), striatum (E), hippocampus (F), amygdala (G), midbrain (H) and cerebellum (I). The activity from 10 to 30 min for each result was magnified on the upper right. Data represent the mean values ± SD (n = 6).


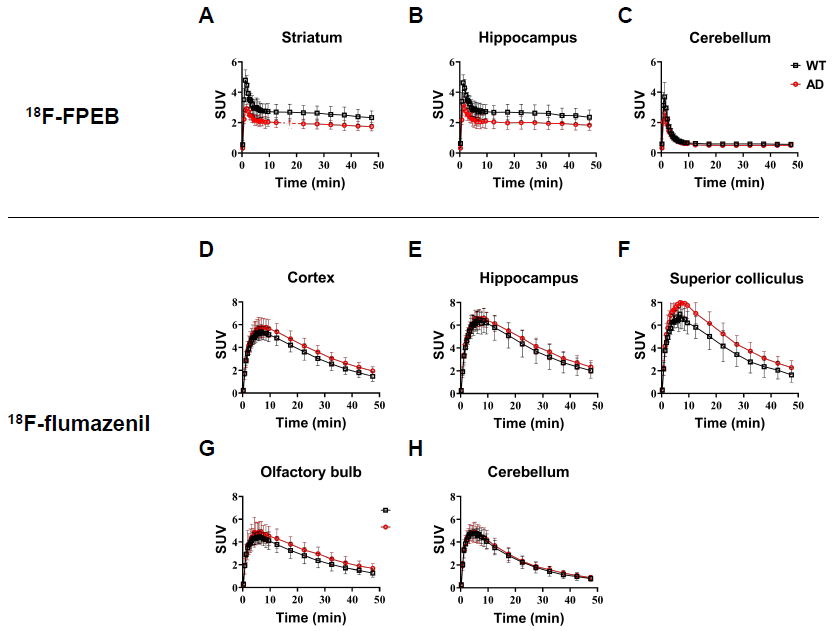


Figure S2. Time-activity curves of target regions for ^18^F-FPEB (A-C) and ^18^F-flumazenil (D-I). Data represent the mean values ± SD (n = 6).


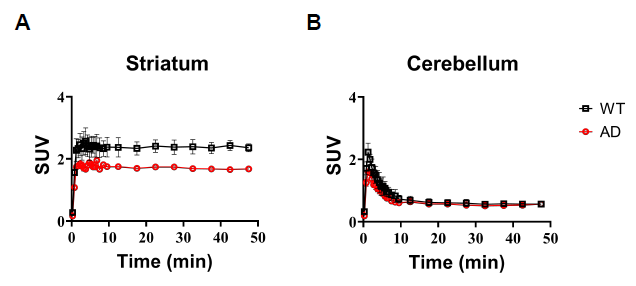


Figure S3. Time-activity curves of the striatum (A) and cerebellum (B) for ^18^F-fallypride. Data represent the mean values ± SD (n = 6).


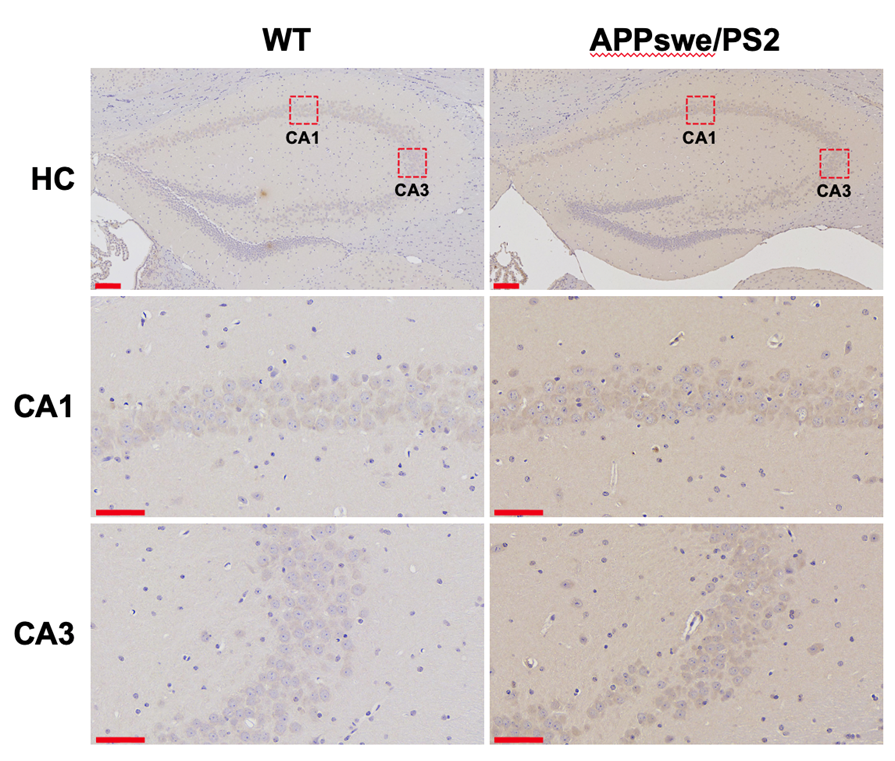


Figure S4. Immunohistochemical staining of Aβ plaque in the hippocampus for WT (left) and APPswe/PS2 mice (right).
